# Preventive effect of ramelteon on emergence agitation after general anaesthesia in paediatric patients undergoing tonsillectomy: a randomised, placebo-controlled clinical trial

**DOI:** 10.1038/s41598-020-79078-4

**Published:** 2020-12-15

**Authors:** Maya Komazaki, Takahiro Mihara, Nobuhito Nakamura, Koui Ka, Takahisa Goto

**Affiliations:** 1 Department of Anesthesiology, Kanagawa Children’s Medical Center, Yokohama, 232-8555 Japan; 2 Department of Anesthesiology and Critical Care Medicine, Yokohama City University Graduate School of Medicine, Yokohama, 236-0004 Japan; 3 Department of Health Data Science, Yokohama City University Graduate School of Data Science, Yokohama, 236-0004 Japan

**Keywords:** Psychiatric disorders, Paediatric research, Clinical trial design, Paediatric research, Preventive medicine

## Abstract

Purpose of this prospective, double-blind, parallel-group, placebo-controlled, randomised clinical trial was to confirm our hypothesis that ramelteon has a preventive effect on emergence agitation after general anaesthesia in children. Patients aged 18 to 119 months (ASA physical status 1 or 2), scheduled to undergo tonsillectomy under general anaesthesia, were randomly allocated to the ramelteon or placebo group. Before general anaesthesia induction, patients in the ramelteon group received 0.1 mg kg^−1^ of ramelteon dissolved in 5 mL of lactose-containing syrup. The patients in the placebo group received the same amount of syrup alone. The Paediatric Anaesthesia Emergence Delirium score was calculated every 5 min after awakening. The primary outcome was the incidence of emergence agitation (Paediatric Anaesthesia Emergence Delirium score ≥ 10). Paediatric Anaesthesia Emergence Delirium scores, post-operative vomiting incidence, pain scores, and adverse events were secondary outcomes. Fifty patients were enrolled. Forty-eight patients were analysed. There was no significant between-group difference in the incidence of emergence agitation (67% in both groups; risk ratio, 1.0; 95% CI 0.67–1.49; *P* > 0.99) or any of the secondary outcomes. Our results suggest that 0.1 mg kg^−1^ of ramelteon does not have a preventive effect on emergence agitation after general anaesthesia in children undergoing tonsillectomy.

## Introduction

Emergence agitation is a common complication after general anaesthesia in children. It is characterised by various manifestations, including crying, excitation, thrashing, disorientation, and incoherence^1,2^. Emergence agitation is a serious problem in paediatric anaesthesia because it increases the risk of self-injury and accidental removal of surgical dressings, intravenous catheters, and drains^3,4^. Moreover, it necessitates extra nursing care and supplemental sedatives or analgesic medications^3^, which can cause respiratory depression. Emergence agitation is encountered more frequently in young patients who undergo tonsillectomy^1,3^. Therefore, preventive measures are particularly necessary for paediatric patients scheduled to undergo tonsillectomy. Some medications such as midazolam^5^, fentanyl^6^, and propofol^7,8^ can be used to prevent or treat emergence agitation; however, the administration of these drugs can result in respiratory depression.

Melatonin is a pineal gland hormone that regulates the sleep–wake rhythm. A previous systematic review and meta-analysis^9^ found that melatonin can prevent emergence agitation without causing respiratory depression in children. Although the point estimate for the effect of melatonin on emergence agitation prevention was strong (risk ratio, 0.31; trial sequential analysis adjusted 95% CI 0.07–1.47), CI for the results was wide; therefore, the authors concluded that additional clinical trials were necessary before firm conclusions could be established.

Ramelteon is a melatonin receptor agonist, and its affinity for the MT1 and MT2 receptors is six and three times higher, respectively, than that of melatonin^10^. Among the various melatonergic agonist drugs, ramelteon has a relatively higher affinity for both receptor subtypes^11^. In addition, the effect of ramelteon on the prevention of delirium in adults has been reported in case series^12–14^, a retrospective study^15^, and randomised controlled trials (RCTs)^16,17^. However, to our knowledge, its preventive effect on emergence agitation in children has not been studied. Therefore, the aim of this randomised, placebo-controlled clinical trial was to confirm our hypothesis that ramelteon has a preventive effect on emergence agitation after general anaesthesia in children.

## Methods

### Setting

This prospective, double-blind, parallel-group, placebo-controlled, randomised clinical trial was approved by the institutional review board of Kanagawa Children’s Medical Center (No. 98–03), Yokohama, Japan (Chairperson: Dr Jiro Machida) on 20 September 2016. The study protocol has been registered in the UMIN Clinical Trial Registry (registration number UMIN000024379, principal investigator: M. Komazaki, Date of registration: 12 October 2016). Written informed consent was obtained from the parents of all patients. The study was conducted in accordance with the principle of the Declaration of Helsinki. An external organisation, Mirai Clinical Trial Support Centre, participated in the protocol review and provided trial oversight and feedback regarding the manuscript. The clinical trial supporting team in our hospital also provided trial oversight and feedback. This study was carried out in accordance with and is reported in adherence to CONSORT guidelines^18^.

### Participants

Patients aged 18 to 119 months (American Society of Anesthesiologists physical status 1 or 2) who were scheduled to undergo tonsillectomy under general anaesthesia were recruited between October 2016 and September 2018. Patients were excluded from the study if they had a history of mental retardation, mental disease, psychotropic or anticonvulsant use, cardiac disease, and/or cerebral surgery. Patients admitted to the hospital in the morning on the day of surgery were also excluded because of the lack of a sufficient time interval between premedication and surgery.

### Randomisation and intervention

The enrolled patients were randomly allocated to a ramelteon or a placebo group. Randomisation was performed using a computer-generated random allocation method (randomization.com). Only the hospital pharmacists were aware of the group allocations, while the patients, parents, anaesthesiologists, outcome assessors, and data collectors were blinded. The study supporter in our hospital confirmed the accuracy of allocation after the surgery.

The enrolled patients were administered ramelteon or placebo 45–60 min before induction, and a pulse oximeter probe was placed on their finger. The patients in the ramelteon group received 0.1 mg kg^−1^ of ramelteon dissolved in 5 ml of syrup containing lactose. The patients in the placebo group received the same amount of syrup alone. We determined the dose of ramelteon according to the previous meta-analysis regarding melatonin^9^, which showed that premedication with 0.1–0.5  mg kg^−1^ of melatonin may effectively prevent emergence agitation^9^. Because ramelteon is a melatonin receptor agonist with higher affinity than that of melatonin itself^10,11^, 0.1 mg kg^−1^ was considered adequate for the present study.

### Anaesthesia

All patients fasted for at least 6 h and were allowed to consume clear fluids for up to 2 h before surgery. On arrival in the operating room, the patients were monitored via non-invasive blood pressure measurements and electrocardiography. Anaesthesia was induced by inhalation of 8% sevoflurane with 4 l min^−1^ nitrous oxide and 2 l min^−1^ oxygen via a face mask; the inhaled and exhaled sevoflurane concentrations were monitored. After loss of consciousness, nitrous oxide was discontinued, the inspired concentration of sevoflurane was reduced to 5% in 100% oxygen, and intravenous access was obtained. Atropine 0.01 mg kg^−1^ and fentanyl 2 μg kg^−1^ were intravenously administered. Rocuronium was also administered if required. After tracheal intubation, the inspired concentration of sevoflurane was reduced to 2–3% in 40% oxygen. Before initiation of the surgery, a paracetamol suppository (30 mg kg^−1^) was administered for post-operative pain control. If the blood pressure or heart rate increased to > 10% above the baseline, fentanyl 1 μg kg^−1^ was administered. After the completion of surgery, the patients were extubated once adequate spontaneous breathing under 100% oxygen with 2–3% end-expiratory sevoflurane was confirmed. If emergence agitation occurred, the anaesthesiologist administered rescue drugs, namely fentanyl, propofol, or droperidol.

### Outcomes and measurement

Before the induction of anaesthesia, the attending anaesthesiologist evaluated the child’s behaviour and anxiety using the Paediatric Anaesthesia Behaviour (PAB) scale^19^. On recovery from anaesthesia in the post-anaesthesia care unit (PACU), the patients were assessed every 5 min using the Paediatric Anaesthesia Emergence Delirium (PAED) scale^2,20^, Aono’s scale^21^, and Children’s Hospital Eastern Ontario Pain Scale (CHEOPS)^22^, and the worst score for each scale was recorded. The PACU nurse or anaesthesiologist, who were blinded to both the group allocation and the PAB score, performed these assessments. The pulse rate and saturation were also monitored during the PACU stay. In addition, we assessed the incidence of post-operative vomiting (POV) during 24 h.

The primary outcome consisted of the incidence of emergence agitation, which was defined by a PAED score of ≥ 10. PAED scores, Aono’s scores, the incidence of POV, CHEOPS scores, the number of patients who required rescue drugs for emergence agitation, PAB scores, the incidence of desaturation in the pre-anaesthesia period and PACU (< 95% in room air), and the time to recovery from anaesthesia in PACU were assessed as secondary outcomes.

### Statistical analysis

According to a previous study conducted at our hospital^23^ and the previous meta-analysis regarding melatonin^9^, we assumed an emergence agitation incidence of 67% and 21% for the placebo and ramelteon groups, respectively. To enable the detection of differences, we set an alpha error of 0.05 and a statistical power of 0.80. Power analysis indicated that the required number of patients in each group was 22. Considering the possibility of dropout, we decided to enrol 50 patients (25 per group).

Analyses were performed for data from a modified intention-to-treat (ITT) population, defined as all randomly assigned patients except those without outcome data.

The Shapiro–Wilk test was used to test the data for normal distribution. Normally distributed continuous data are presented as mean ± SD and were analysed using the unpaired t-test. Non-normally distributed data are presented as median [IQR] and were analysed using the Mann–Whitney *U* test. Categorical variables are presented as number (%) and were analysed using Fisher’s exact test. We calculated mean differences, median differences, or risk ratios with 95% CIs. 95% CIs for median differences were calculated using the bootstrap re-sampling method (2000 samples). A *P* value of < 0.05 was considered statistically significant. All statistical analyses were performed using R 3.4.3 (The R Project for Statistical Computing, November 2017; www.r-project.org).

## Results

**Figure 1:**
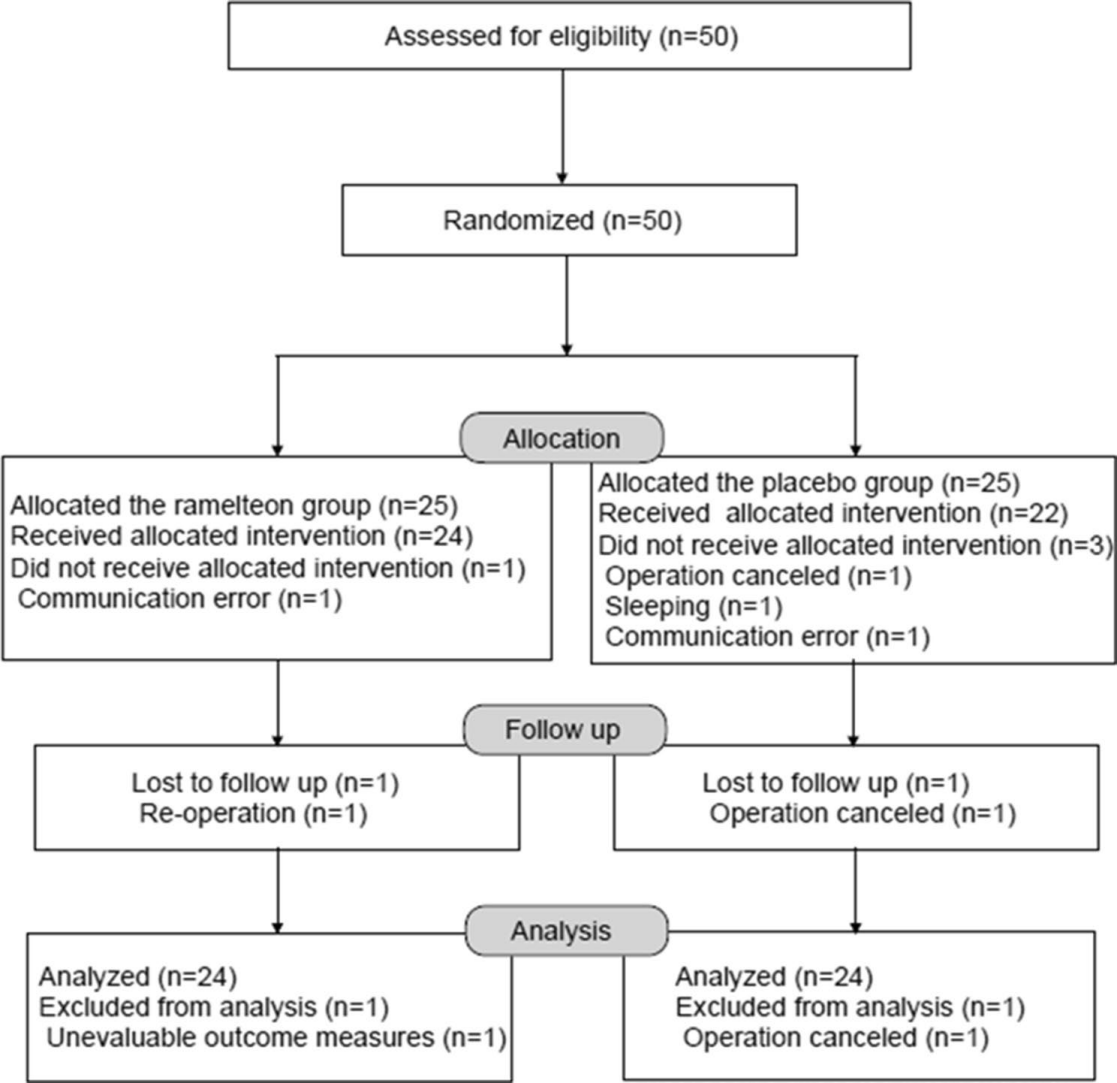
Flow diagram showing the selection of patients for a randomised, placebo-controlled clinical trial on the preventive effects of ramelteon on emergence agitation in children.

We recruited patients until the target sample size was achieved. Fifty patients were enrolled, and two were excluded after randomisation because of surgery cancellation for one patient in the placebo group and lack of assessment of the primary outcome for one patient in the ramelteon group, who developed bleeding on arrival in PACU and was taken back to the operating room. Eventually, 48 patients were eligible (Fig. 1). Three patients did not receive premedication; one patient each in the placebo and ramelteon groups was not prescribed the study drug because of our communication error, while one patient in the placebo group was sleeping at the time of premedication. These three patients were included in the modified ITT analysis.

### Patient characteristics

Patient demographics, anaesthesia and surgical durations, and total fentanyl doses are presented in Table 1. The mean patient age was 63.4 months, and the median body weight was 16.7 kg. The mean surgical duration was < 1 h. There were no significant differences in any parameter between the two groups.

**Table 1:** Characteristics of paediatric patients who received ramelteon or placebo for the prevention of emergence agitation. Data are presented as mean ± SD, median [IQR], or number.

	Placebo (n = 24)	Ramelteon (n = 24)
Sex; male (%)	19 (79.2)	18 (75.0)
Age (months)	61.9 ± 23.1	64.9 ± 22.9
ASA-PS	2 [1 to 2]	2 [1 to 2]
Height (cm)	105 ± 14.6	107 ± 14.7
Weight (kg)	16.6 [14.0 to 20.2]	17.2 [14.0 to 20.0]
Anaesthesia duration (min)	93 ± 21	87 ± 18
Surgical duration (min)	52 ± 16.0	48 ± 14.1
Total fentanyl dose (μg kg^−1^)	5.6 [4.3 to 6.6]	4.7 [4.0 to 6.0]

ASA-PS: American Society of Anesthesiologists Physical Status.

### Primary and secondary outcomes

There was no significant difference in the incidence of emergence agitation between the two groups (67% in both groups; risk ratio, 1.0; 95% CI 0.67–1.49; *P* > 0.99; Table 2. Moreover, the median PAED [13.5 (IQR 7.5 to 18.0) vs. 13.5 (IQR 6.8 to 19.2) in the placebo and ramelteon groups; *P* = 0.79] and Aono’s scores [3 (IQR 1 to 4) vs. 3 (IQR 1.75 to 4) in the placebo and ramelteon groups; *P* = 0.80] were comparable between groups. The incidence of POV, number of patients who required rescue drugs for emergence agitation, and median CHEOPS and PAB scores (Table 2) also showed no significant intergroup differences. The incidence of desaturation in PACU was 25% (6/24) and 4.2% (1/24) in the placebo and ramelteon groups, respectively (*P* = 0.10), while the time to recovery from anaesthesia in PACU was 16 (12 to 22) and 12 (7 to 18) min, respectively (*P* = 0.08). One patient in the placebo group exhibited desaturation (94% in room air) in the pre-anaesthesia period.

**Table 2:** Primary and secondary outcomes for paediatric patients who received ramelteon or placebo for the prevention of emergence agitation. Data are presented as number or median [IQR].

	Placebo (n = 24)	Ramelteon (n = 24)	Risk ratio or median difference (95% CI)	*P* value
Agitation (%)	16 (66.7)	16 (66.7)	1.0 (0.67 to 1.49)	> 0.99
PAED score	13.5 [7.5 to 18.0]	13.5 [6.8 to 19.2]	0 (− 4.8 to 5.6)*	0.79
Aono’s score	3 [1 to 4]	3 [1.75 to 4]	0 (− 1.1 to 1.4)*	0.80
Rescue drug use (%)	11 (45.8)	13 (54.2)	1.18 (0.67 to 2.09)	0.77
Desaturation in PACU (%)	6 (25.0)	1 (4.2)	0.17 (0.02 to 1.28)	0.10
POV (%)	6 (25.0)	5 (20.8)	0.83 (0.29 to 2.37)	> 0.99
Time to recovery from anaesthesia (min)	16 [12 to 22]	12 [7 to 18]	− 4.0 (− 9.1 to 0.1)*	0.08
CHEOPS score	12.0 [7.5 to 13]	11.5 [9 to 12]	− 0.5 (− 2.5 to 1.6)*	0.56
PAB score	1 [1 to 2]	1 [1 to 2]	0 (− 0.6 to 0.9)*	0.77

PAED: Paediatric Anaesthesia Emergence Delirium, PACU: post-anaesthesia care unit, POV: post-operative vomiting, CHEOPS: Children’s Hospital Eastern Ontario Pain Scale, PAB: Paediatric Anaesthesia Behaviour.

*The 95% confidence interval of the median difference was calculated using the bootstrap method.

## Discussion

Our results revealed that premedication with 0.1 mg kg^−1^ of ramelteon premedication did not have a preventive effect on emergence agitation after general anaesthesia in children undergoing tonsillectomy. Not only the incidence of emergence agitation but also the PAED and Aono’s scores were similar in the placebo and ramelteon groups. In addition, ramelteon showed no beneficial effects on pain, the incidence of POV, and the pre-operative behaviour, and it did not delay recovery from anaesthesia or increase the incidence of desaturation in PACU. Thus, our hypothesis was rejected.

A previous systematic review and meta-analysis^9^ showed that 0.1–0.5 mg kg^−1^ of melatonin has a preventive effect on emergence agitation in a dose-dependent manner, with a stronger preventive effect at a higher dose (0.5 mg kg^−1^) than at a lower dose (0.1 mg kg^−1^). The affinity of ramelteon for the MT1 and MT2 receptors is six and three times higher, respectively, than that of melatonin^10^; therefore, we considered that 0.1 mg kg^−1^ of ramelteon would be enough to prevent emergence agitation. Although our results clearly showed that this dose of ramelteon does not prevent emergence agitation in children, further studies should confirm the preventive effects of high-dose ramelteon on emergence agitation in children.

In the present study, pain scores (CHEOPS score), pre-operative behaviour scores (PAB score), and the incidence of POV were comparable between the placebo and ramelteon groups. Therefore, 0.1 mg kg^−1^ of ramelteon is not recommended for reducing the post-operative pain intensity or preventing POV in children undergoing tonsillectomy. The results of the PAB score, however, should be interpreted with caution. Considering the low score in the control group, a beneficial effect of ramelteon on the PAB score could not be demonstrated in our study setting. The anti-nociceptive effect of melatonin is controversial. While some clinical trials^24–26^ and systematic reviews^27,28^ reported that melatonin premedication decreases post-operative pain relative to that experienced with placebo, other clinical trials^29–32^ showed that melatonin does not alter pain. Our results showed that 0.1 mg of ramelteon has no relieving effect on post-operative pain in children undergoing tonsillectomy.

We also confirmed that 0.1 mg kg^−1^ of ramelteon did not increase the incidence of desaturation throughout the study period. Patients undergoing tonsillectomy often exhibit problems such as snoring and obstructive sleep apnoea^33–35^. Thus, we started respiratory monitoring from the time of ramelteon administration in the ward, and there was no increase in the incidence of respiratory depression, consistent with the findings in the previous meta-analysis regarding melatonin. However, our results cannot be extrapolated to patients who receive a dose higher than 0.1 mg kg^−1^, and we recommend that respiratory monitoring should be initiated from the time of ramelteon administration in future trials investigating higher doses.

This study has several strengths. First, only the pharmacists had the randomised number list and were aware of the allocation groups. Therefore, allocation concealment was maintained throughout the study period. Second, we could blind the surgeons, nurses, anaesthesiologists, data collectors, outcome evaluators, patients, and parents to the allocated treatment, which helped in controlling information bias.

This study also has a number of limitations. First, our study was conducted as a single-centre design, and we only included patients requiring tonsillectomy, which is associated with a high risk of emergence agitation. Thus, we cannot apply our results to procedures performed at other centres and patients undergoing other surgeries. The four studies in the previous systematic review^9^ did not include patients undergoing tonsillectomy, and their incidence of emergence agitation in the control group (i.e., 27 to 50%) was lower than ours. The patients who underwent tonsillectomy might experience pain and a sense of suffocation, thereby contributing to emergence agitation^1,3^. The high risk of emergence agitation in a tonsillectomy might affect our negative results. Although our study showed that ramelteon had no preventive effect on emergence agitation in patients undergoing a tonsillectomy, our results cannot be extrapolated to other surgeries. Second, we assessed 0.1 mg kg^−1^ ramelteon only. Although we decided on the ramelteon dose based on its high affinity to the MT1 and MT2 receptors^10,11^, the high affinity may not necessarily mean that the effect is greater. Thus, there remains a possibility that a higher dose of ramelteon may be effective. Third, this study included patients aged 18 to 119 months. Psychological development depends on patients' age, and the difference in psychological development may affect the outcomes. However, we could not assess the effect among different age groups because our trial includes a small number of patients.

In conclusion, ramelteon at a dose of 0.1 mg kg^−1^ does not prevent emergence agitation after general anaesthesia in paediatric patients undergoing tonsillectomy, and it also does not prolong the time to recovery from anaesthesia or increase the incidence of desaturation in PACU. Further studies should investigate the effects of high-dose ramelteon on emergence agitation in children.

## Data Availability

The datasets generated and/or analysed during the current study are available from the corresponding author upon reasonable request.
